# Long-term oncological outcomes after laparoscopic parenchyma-sparing redo liver resections for patients with metastatic colorectal cancer: a European multi-center study

**DOI:** 10.1007/s00464-021-08655-z

**Published:** 2021-08-30

**Authors:** Leonid Barkhatov, Davit L. Aghayan, Vincenzo Scuderi, Federica Cipriani, Åsmund A. Fretland, Airazat M. Kazaryan, Francesca Ratti, Thomas Armstrong, Andrea Belli, Ibrahim Dagher, Giulio Belli, Luca Aldrighetti, Mohammad Abu Hilal, Roberto I. Troisi, Bjørn Edwin

**Affiliations:** 1 The Intervention Centre, Oslo University Hospital, Rikshospitalet, Norway; 2 Department of Acute and Digestive Surgery, Haukeland University Hospital, Bergen, Norway; 3 Institute of Clinical Medicine, Oslo University, Rikshospitalet, Norway; 4 Department of General Surgery, Pellegrini Hospital, Naples, Italy; 5 Hepatobiliary Surgery Division, IRCCS San Raffaele Scientific Institute, Milan, Italy; 6 Department of Hepatic, Pancreatic and Biliary Surgery, Oslo University Hospital, Rikshospitalet, Norway; 7 Department of Digestive Surgery, Østfold Hospital Trust, Grålum, Norway; 8 Department of Minimally Invasive Digestive Surgery, Antoine-Beclere Hospital, Assistance Publique - Hôpitaux de Paris, Clamart, France; 9 Paris-Saclay University, Orsay, France; 10 Department of General and HPB Surgery, S.M. Loreto Nuovo Hospital, Naples, Italy; 11 Department of Surgery, Southampton University Hospital NHS Foundation Trust, Southampton, UK; 12 Division of HPB, Department of Clinical Medicine and Surgery, Minimally Invasive and Robotic Surgery, Federico II University Hospital, Naples, Italy; 13 Vita-Salute San Raffaele University, Milan, Italy; 14 Department of Surgery, Fondazione Poliambulanza - Instituto Ospedaliero, Brescia, Italy; 15 Department of Abdominal Oncology, HPB Surgical Oncology Unit, National Cancer Institute, Fondazione G. Pascale - IRCCS, Naples, Italy

**Keywords:** Laparoscopic liver surgery, Redo liver resections, Colorectal liver metastases, Parenchyma-sparing liver resections

## Abstract

**Background:**

Laparoscopic redo resections for colorectal metastases are poorly investigated. This study aims to explore long-term results after second, third, and fourth resections.

**Material and methods:**

Prospectively updated databases of primary and redo laparoscopic liver resections in six European HPB centers were analyzed. Procedure-related overall survival after first, second, third, and fourth resections were evaluated. Furthermore, patients without liver recurrence after first liver resection were compared to those with one redo, two or three redo, and patients with palliative treatment for liver recurrence after first laparoscopic liver surgery. Survival was calculated both from the date of the first liver resection and from the date of the actual liver resection. In total, 837 laparoscopic primary and redo liver resections performed in 762 patients were included (630 primary, 172 first redo, 29 second redo, and 6 third redo). Patients were bunched into four groups: Group 1—without hepatic recurrence after primary liver resection (*n* = 441); Group 2—with liver recurrence who underwent only one laparoscopic redo resection (*n* = 154); Group 3—with liver recurrence who underwent two laparoscopic redo resections (*n* = 29); Group 4—with liver recurrence who have not been found suitable for redo resections (*n* = 138).

**Results:**

No significant difference has been found between the groups in terms of baseline characteristics and surgical outcomes. Rate of positive resection margin was higher in the group with palliative recurrence (group 4). Five-year survival calculated from the first liver resection was 67%, 62%, 84%, and 7% for group 1, 2, 3, and 4, respectively. Procedure-specific 5-year overall survival was 50% after primary laparoscopic liver resection, 52% after the 1st reoperation, 52% after the 2nd, and 40% after the 3rd reoperation made laparoscopic.

**Conclusions:**

Multiple redo recurrences can be performed laparoscopically with good long-term results. Liver recurrence does not aggravate prognosis as long as the patient is suitable for reoperation.

Liver is one of the most frequent sites for recurrence of colorectal cancer, with a 50–60% rate of spread to the liver after primary colorectal surgery [1, 2]. Liver resections increase survival dramatically, making surgery the only curative option for patients with liver metastases [3–5]. However, up to 50–60% of patients experience liver recurrence after primary liver surgery, and redo resections are technically more demanding due to anatomical changes, postoperative adhesions, and a risk of liver insufficiency [6].

Laparoscopic technique for liver resections has become an increasing trend in the past decade in the specialized hepatobiliary centers [7]. Laparoscopy gives potential advantages in reduced morbidity, reduced hospital stay, and a reduction in severity of postoperative adhesions, with no negative influence on long-term survival [8–11].

One of the important surgical options for liver metastases is atypical parenchyma-sparing resections. Parenchyma-sparing resections, unlike anatomical resections, have been widely accepted both for laparoscopic and open access [12–16]. Preservation of liver parenchyma decreases risk of insufficiency and can make redo resections more feasible in the case of recurrence. Furthermore, an animal trial demonstrated that major resections can be associated with increased development of tumor growth compared to minor resections [17].

In the era of modern chemotherapy regimes, repeated resections for liver recurrences are possible and show favorable results [18–21]. Despite the good result after open redo hepatectomies, the long-term outcomes of laparoscopic resections for liver recurrences are still poorly reported [22–24].

The aim of this study was to analyze oncological outcomes for patients who underwent laparoscopic reoperation for repeated liver recurrence of colorectal cancer.

## Material and method

The data were collected from six European centers with a large expertise in laparoscopic hepato-pancreato-biliary surgery: Oslo University Hospital (Oslo, Norway), Ghent University Hospital (Ghent, Belgium), University Hospital Southampton (Southampton, United Kingdom), San Raffaele Hospital (Milan, Italy), Antoine-Beclere Hospital (Paris, France), and S. M. Loreto Nuovo Hospital (Naples, Italy).

The data collected included intra- and postoperative parameters. Survival was calculated both from the date of the first liver resection, and from the date of the actual liver resection.

All patients that underwent laparoscopic liver resections (LLR) for primary or recurrent colorectal liver metastases from the beginning of laparoscopic HPB surgery in each center until February 2016 were included in the study. Patients who underwent two-stage liver resection, RF ablation combined with liver resection, and patients who underwent previous liver transplantation were excluded.

The surgical technique has been described earlier for each center [24–29]. Parenchyma-sparing resections have been applied whenever technically feasible. The preoperative workup for LLR was similar for open and laparoscopic liver resections (clinical biochemistry, ultrasonography, spiral CT, magnetic resonance imaging, and positron emission tomography-CT when required).

All patients were discussed at a multidisciplinary team meeting that included surgeons, radiologists, and oncologists before the decision to operate was made.

Continuous perioperative data were reported as median (ranges). Survival analysis was performed using Kaplan–Mayer method. Log-rank test was applied for comparison of survival between groups. Survival length was presented as median (95% confidential interval). Overall survival was calculated from the time of the primary liver resection in each group of patients, as well as from the time of actual redo resection (procedure-related overall survival).

The study has been approved by Institutional Data Protection Official. Written consent from the patients was not needed because of the retrospective nature of the study.

## Results

We found 860 laparoscopic primary and redo liver resections that met the inclusion criteria. A total of 787 patients underwent surgery, but 25 were excluded from analysis (two patients due to previous liver transplantation, 13 patients with combined RF ablations, and ten patients underwent two-stage liver resection). Therefore, 762 patients and 837 procedures constitute the foundation of the study. Of these, 630 resections were performed as a primary procedure, while 172 were first redo, 29 were second, and six were third redo resections.

Patients were divided into four groups: Group 1—patients without hepatic recurrence after primary liver resection (*n* = 441); Group 2—patients with liver recurrence who underwent one laparoscopic redo resection during the observation period (*n* = 154); Group 3—patients with liver recurrence who underwent two or three laparoscopic redo resections during the observation period (*n* = 29); Group 4—patients with liver recurrence who were not found suitable for a redo resections (*n* = 138).

Preoperative characteristics are presented in Table 1. No significant difference was found between the groups in terms of age, gender, BMI, tumor localization, chemotherapy, number of tumors, and tumor size.

**Table 1:** Demographic and preoperative parameters

	Group 1 *n* = 441	Group 2 *n* = 154	Group 3 *n* = 29	Group 4 *n* = 138	*p* value
Age, years, median	67 (29–89)	66 (32–86)	63 (43–83)	68 (37–86)	n/s
Male gender, %	58%	57%	46%	55%	n/s
BMI, median (range)	25 (15–57)	25 (18–50)	26 (16–32)	25 (19–38)	n/s
ASA score, median (range)	2 (1–3)	2 (1–3)	2 (2–3)	2 (1–3)	n/s
Type of previous liver resection(s), %					
Posterosuperior segments (n)	n/a	40% (62)	45% (13)	n/a	
Formal left/right (n)		9% (14)	31% (9)		
Anterolateral segments (n)		51% (78)	24% (7)		
Tumor localization, %					
Left lobe (n)	27% (119)	43% (66)	56% (16)	29% (40)	
Right lobe (n)	49% (216)	47% (72)	36% (11)	40% (55)	
Bilateral (n)	24% (106)	10% (16)	8% (2)	31% (43)	
Neoadjuvant chemotherapy	37%	44%	33%	50%	n/s
Tumor size, median, mm (range)	25 (2–120)	25 (6–120)	22 (10–50)	30 (1–100)	n/s
N of tumors, median	1 (1–7)	1 (1–12)	1 (1–3)	1 (1–10)	n/s
Total N of liver procedures	1	2	3 (3–4)	1	0.001

Intra- and postoperative results are presented in Table 2 for group 1, 2, 3, and 4, and in Table 3 for 1st, 2nd, 3rd, and 4th recurrences. There was no significant difference in terms of operation time, bleeding, hemotransfusion, conversion rate, postoperative complications, or hospital stay. Higher rate of positive resection margin (R1) has been found in group 4, when compared to groups 1 and 2.

**Table 2:** Intra- and postoperative parameters; group analyses

	Group 1 *n* = 441	Group 2 *n* = 154	Group 3 *n* = 29	Group 4 *n* = 138	*p* value
*N* of resected specimens, median	1 (1–5)	1 (1–3)	1 (1–5)	1 (1–5)	n/s
Resection margin, median, mm	4.0 (0–50)	3.0 (0–17)	3.0 (0–10)	2.0 (0–30)	n/s
Resection status					
R0	89%	90%	84%	73%	< 0.001*
R1	11%	10%	16%	27%	
Operation time, median, min	131 (25–635)	185 (28–540)	185 (25–570)	160 (21–430)	n/s
Bleeding, median, ml	200 (10–4000)	200 (10–2600)	100 (10–5000)	250 (10–3000)	n/s
Hemotransfusion, %	12%	8.6%	12%	15%	n/s
Conversion, %	3%	9%	16%	3%	
Open	2%	4.5%	12%	3%	
HALS	1%	4.5%	4%	0%	
LoS	3 (1–33)	3 (1–50)	2 (1–8)	3 (1–26)	
Post-operative complications, %					
Minor	13%	14%	15%	12%	n/s
Major	5%	6%	5%	5%	n/s

*p value < 0.001 between the groups 1 and 4, and groups 2 and 4; non-significant between the groups 1 and 2, 1 and 3, 2 and 3, 3 and 4

**Table 3:** Intra- and postoperative parameters for 1st, 2nd, 3rd, and 4th liver resections

	1st resection *n* = 630	2nd resection *n* = 172	3rd resection *n* = 29	4th resection *n* = 6	*p* value
*N* of resected specimens, median	1 (1–6)	1 (1–3)	1 (1–5)	1 (1–1)	n/s
Resection margin, median, mm	4.0 (0–65)	3.0 (0–19)	3.0 (0–10)	2.0 (0–5)	n/s
Resection status					
R0	87%	89%	84%	33%	n/s
R1	13%	11%	16%	66%	
Operation time, median, min	135 (21–635)	210 (36–540)	185 (25–570)	203 (83–390)	n/s
Bleeding, median, ml	220 (10–5000)	200 (10–2500)	100 (10–5000)	190 (10–800)	n/s
Hemotransfusion, %	12%	7%	12%	0%	n/s
Conversion, %	4%	14%	16%	50%	
Open	2%	9%	12%	33%	
HALS	2%	5%	4%	17%	
LoS	3 (1–33)	3 (1–50)	2 (1–8)	2 (2–8)	n/s
Post-operative complications, %					
Minor	13%	14%	15%	33%	n/s
Major	8%	7%	5%	0%	

### Oncological results

**Fig. 1:**
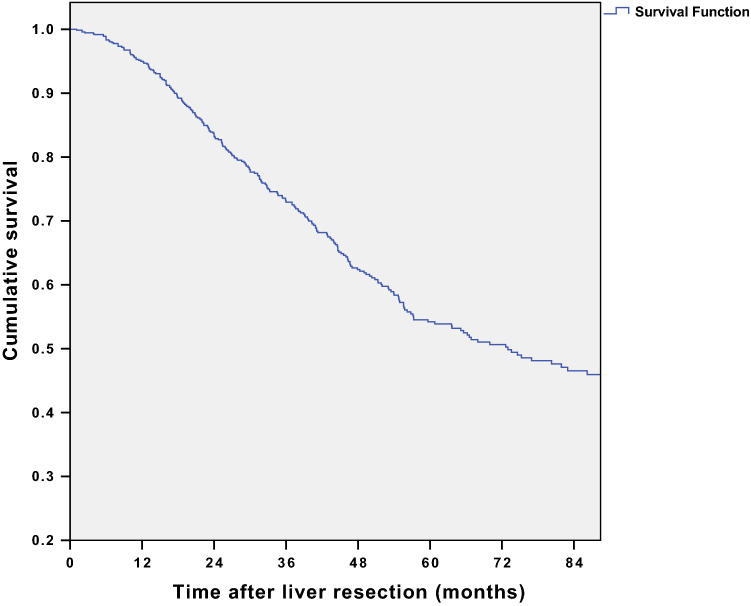
Overall survival for the total cohort, *n* = 762 patients

Overall survival for the whole cohort of 762 patients is presented in Fig. 1. 1-, 3-, and 5-year survival was 95%, 73%, and 54%, respectively, with median survival of 73 ± 15.9 (95% CI) month.

**Fig. 2:**
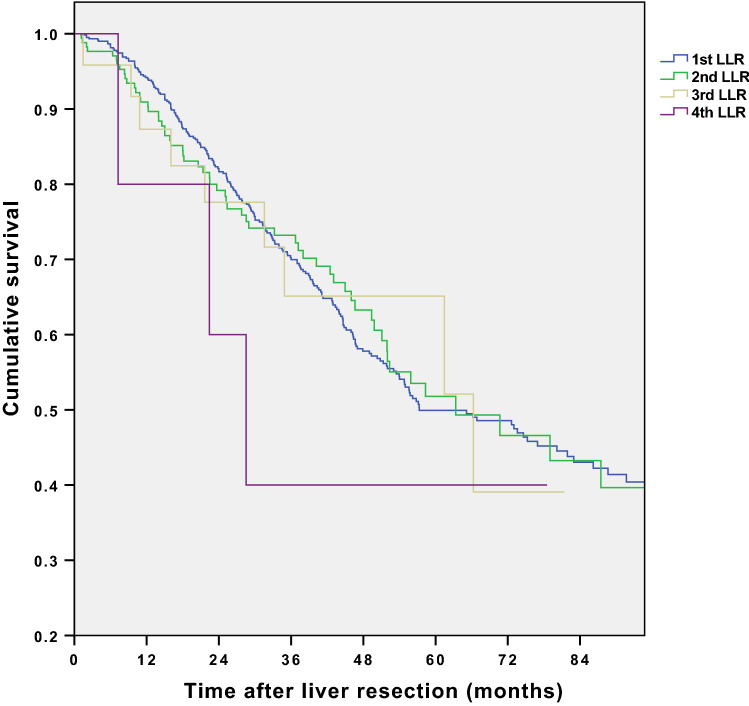
Procedure-related overall survival for the first (1), second (2), third (3), and fourth (4) LLR. Survival has been calculated from the time of actual liver resection

Figure 2 demonstrates procedure-specific overall survival. Line 1 represents primary laparoscopic liver resection (*n* = 630), line 2—the first reoperation (*n* = 172), line 3—second reoperation (*n* = 27), and line 4—third reoperation done laparoscopically (*n* = 6). 1-, 3- and 5-year survival was 94%, 70%, and 50% in group 1; 90%, 72%, and 52% in group 2; 87%, 65%, and 52% in group 3; and 80%, 40%, and 40% in group 4, respectively. The median length of survival was 57.3 ± 13.2 (95% CI), 63.4 ± 24.5 (95% CI), 66.3 ± 37.1 (95% CI), and 28.5 ± 13.1 (95% CI) month, respectively.

**Fig. 3:**
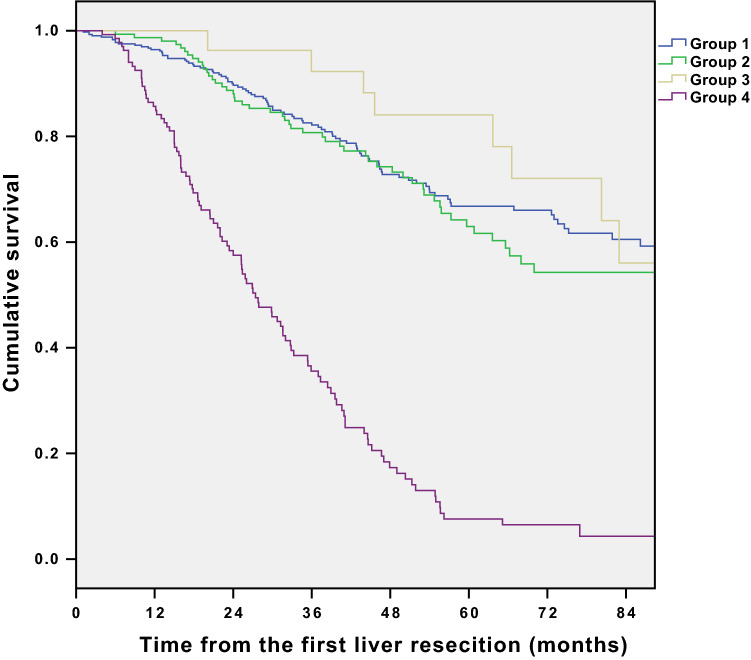
Overall survival for patients with: 1—one LLR without liver recurrence; 2—only one redo LLR; 3—two or three redo LLR; 4—one LLR and no surgical treatment for liver recurrence. Survival has been calculated from the time of the first liver resection

For the group analysis, overall survival has been presented in Fig. 3. Survival has been calculated from the time of the primary liver resection. 1-, 3-, and 5-year survival was 96%, 82%, and 67% in group 1; 98%, 80%, and 62% in group 2; 96%, 92, and 84% in group 3; and 84%, 35%, and 7% in group 4, respectively. Median length of survival was 109.7 ± 28.5 (95% CI), 98.7 ± 37.8 (95% CI), 109.9 ± 58.2 (95% CI), and 27.4 ± 4.5 (95% CI) month, respectively.

## Discussion

In this study, we analyzed long-term oncological results in patients who underwent laparoscopic resection for recurrent colorectal liver metastases. For selected groups of patients who had one or more resectable liver recurrences and was suitable for redo operation, survival seems to be similar to that in patients who did not have a liver recurrence after the primary liver resection. However, statistical comparison of survival in these groups is not possible due to immortal time bias. Also, procedure-related survival for first, second, and third liver resections seems to be similar. Laparoscopy as fourth liver resection is feasible and shows sufficient survival, even so this group consist of only six patients, and result should be interpreted with caution.

Most of redo resections in this study were performed using atypical parenchyma-sparing or minor resections. While hemihepatectomy can be an efficient and radical solution for several unilobar metastases, it leaves little room for future maneuvers if further recurrences arise.

Despite the technical challenge of repeated resections, a significant difference in intraoperative results after redo resections comparing with primary resections was not found, notwithstanding the fact that all patients had similar preoperative parameters. Interestingly, patients who had unresectable recurrence after the first LLR (Group 4) had higher rate of R1 resection margin. This might have affected survival in this group.

In a meta-analysis of eight studies, Wurster et al. showed similar morbidity and survival for repeated open resections of colorectal metastases, with no significant difference between single and repeated resections [18]. Also, some of the studies reported better survival for patients with repeated resections compared to those with single liver resections [30–32]. Favorable oncological outcomes using the laparoscopic approach with a parenchyma-preserving surgery eventually increasing the number of iterative procedures were demonstrated in a case–control matched-pairs analysis [26]. However, this improved outcome is most likely related to selection, and this also reflects our experience where some patients in the single resection group had non-operable recurrences. To minimize these selection biases, we divided patients with only primary resections into two groups: those who did not have a liver recurrence, and those who had a recurrence and were deemed as palliative patients. Survival for patients after 2nd and 3rd liver resections was comparable for those with primary resections without recurrence.

OSLO-COMET randomized controlled trial demonstrated that laparoscopic liver resections decrease postoperative complications and the length of postoperative hospital stay compared to open technique [10]. A shorter recovery time is even more important for patients who require several reoperations—a decrease in occurrence of abdominal infections and major complications leads to a decrease in postoperative adhesions. Thus, a shorter reconvalescence time enables an earlier start of adjuvant chemotherapy and makes operation/reoperation for extrahepatic metastases possible sooner. For patients with a constantly present oncologic illness that require several reoperations, a decrease in length of hospital is a notable factor, together with an improved quality of life compared to that resulting from open hepatectomies [33]. Laparoscopic approach has the added benefit that it causes less postoperative adhesions, which, in turn, makes further reoperations easier and more feasible [34].

Furthermore, when several reoperations are needed, choosing laparoscopic approach compared to open reoperation can keep the trauma-induced postoperative inflammatory response to a minimum [35], and might hypothetically reduce the risk factors for tumor metastatic formation.

This study was based on retrospective data, which may lead to heterogeneity and performance bias. To minimize those biases, a prospective-updated database of consecutive cases was collected for each center. Also, preoperative characteristics and post-operative results were analyzed in order to minimize performance bias. However, performing an RCT comparing redo with singular resections is nearly impossible because of ethical reasons.

Another limitation is the multi-center nature of the study, which may be considered a weakness due to the potential heterogeneity of patients’ groups and differences in surgical routines and follow-up, but also as a benefit due to higher reproducibility of the study.

## Conclusion

Our results demonstrate that liver recurrence does not aggravate prognosis as long as the patient is suitable for reoperation. Surgery provides clear benefits even for 2nd, 3rd, and 4th recurrences if the recurrence is technically suitable for radical resection. For patients with several re-resections, the benefit of parenchyma-sparing technique and laparoscopy can increase with each subsequent resection, with favorable long-term outcomes.
